# Insidious intraoperative ureteral injury as a complication in oblique lumbar interbody fusion surgery: a case report

**DOI:** 10.1186/s13104-017-2509-9

**Published:** 2017-06-06

**Authors:** Go Kubota, Sumihisa Orita, Tomotaka Umimura, Kazuhisa Takahashi, Seiji Ohtori

**Affiliations:** Department of Orthopedic Surgery, Graduate School of Medicine, Chiba University, 1-8-1 Inohana, Chuo-Ku, Chiba, 260-8677 Japan

**Keywords:** Oblique lumbar interbody fusion (OLIF), Minimally invasive surgery, Lumbar spine, Ureteral injury, Complications, Interbody fusion, Retroperitoneal approach

## Abstract

**Background:**

Oblique lumbar interbody fusion surgery is a recently introduced minimally invasive lateral interbody fusion surgery for degenerative lumbar disease. There have been no reports of associated ureteral injury.

**Case presentation:**

A 77-year-old Japanese woman underwent oblique lumbar interbody fusion surgery for lumbar spondylolisthesis with refractory low back pain and pain in both legs. The patient experienced abdominal pain 2 days after surgery. Delayed contrast-enhanced computed tomography and retrograde urography revealed leakage of contrasted urine from the ureter into the retroperitoneal space, indicating a ureteral injury. Immediate percutaneous nephrostomy was performed to recover her condition, followed by additional ureteral stenting. She is now free from preoperative symptoms but requires periodic changing of the ureteral stent, with no urinary symptoms.

**Conclusion:**

The current report described a rare but possible case of ureteral injury following oblique lumbar interbody fusion surgery. Iatrogenic ureteral injury, as reported in the current case, is uncommon following oblique lumbar interbody fusion surgery, and the injury may have been caused by a procedural error. Considering the findings from urological examinations, we speculate that the thread pin that fixates the retractor injured the ureter during its installation. This case highlights the importance of careful attention while exposing the retroperitoneal space to avoid minor organs, including the ureters, as well as major organs. Ureteral injuries should ideally be detected and diagnosed as soon as possible by careful physical and radiological examinations, such as with delayed contrast-enhanced computed tomography and retrograde urography, to salvage the injured nephroureteral system. The current report also highlights that careful use of surgical instruments is key for preventing intraoperative complications, including ureteral injury.

## Background

Oblique lateral interbody fusion (OLIF) surgery is a minimally invasive lateral lumbar interbody fusion surgery for degenerative lumbar diseases, such as spondylolisthesis and kyphoscoliosis [1–5]. The procedure is safer and more effective than the traditional spinal fusion surgeries in this patient population, although some intraoperative complications can occur. To the limited number of reported complications following this procedure [2], we add a case of intraoperative ureteral injury, followed by a discussion of its predisposing factors and clinical characteristics.

## Case presentation

**Fig. 1:**
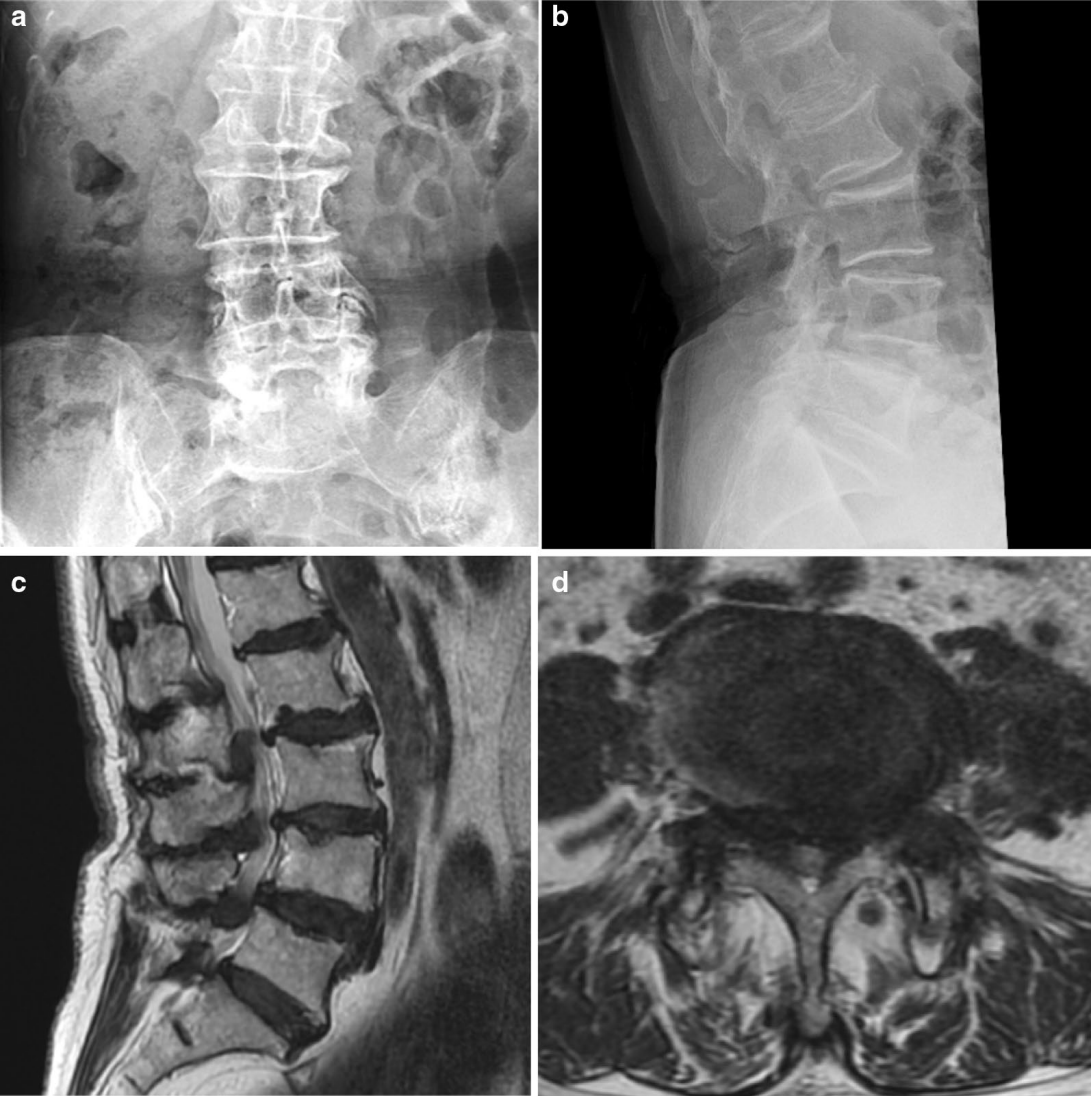
Preoperative radiography (**a**, **b**) and magnetic resonance imaging (**c**, **d**) revealed L4/5 lumbar spinal stenosis

**Fig. 2:**
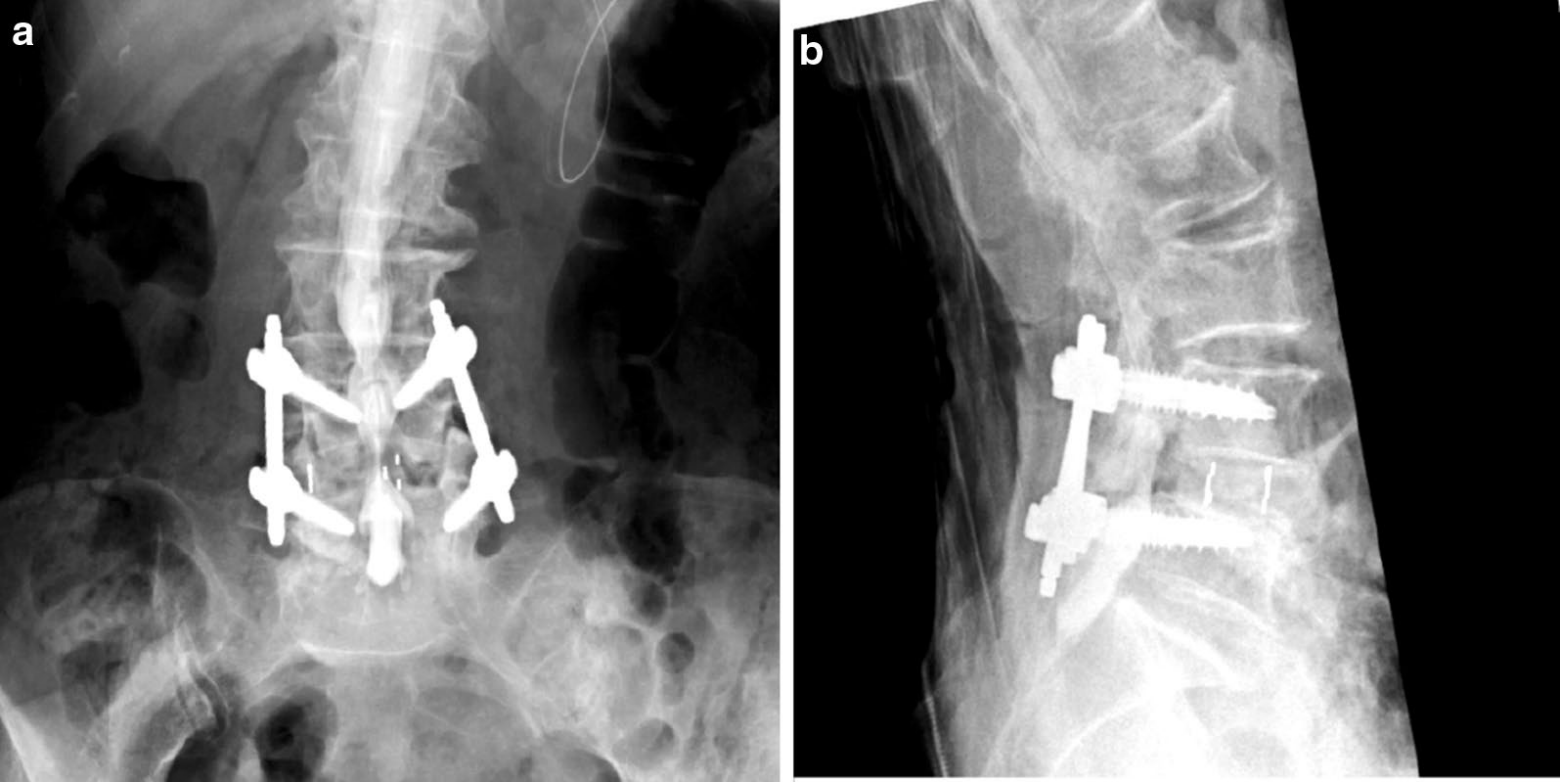
Postsurgical radiography. **a** Antero-posterior view. **b** Lateral view

A 77-year-old Japanese woman with rheumatoid arthritis (RA) was diagnosed with lumbar spinal stenosis. She underwent OLIF surgery followed by posterior fusion using percutaneous pedicle screws at our department for refractory bilateral buttock pain and sciatica (Fig. 1). The course of surgery was smooth except for one issue: when placing the self-retaining retractor onto the L4–5 intervertebral disc, a thread pin, which fixates the retractor through a small hole, went deep into the anterior space of the spinal column. Postoperative plain radiography showed adequate stability with recovered disc height (Fig. 2).

**Fig. 3:**
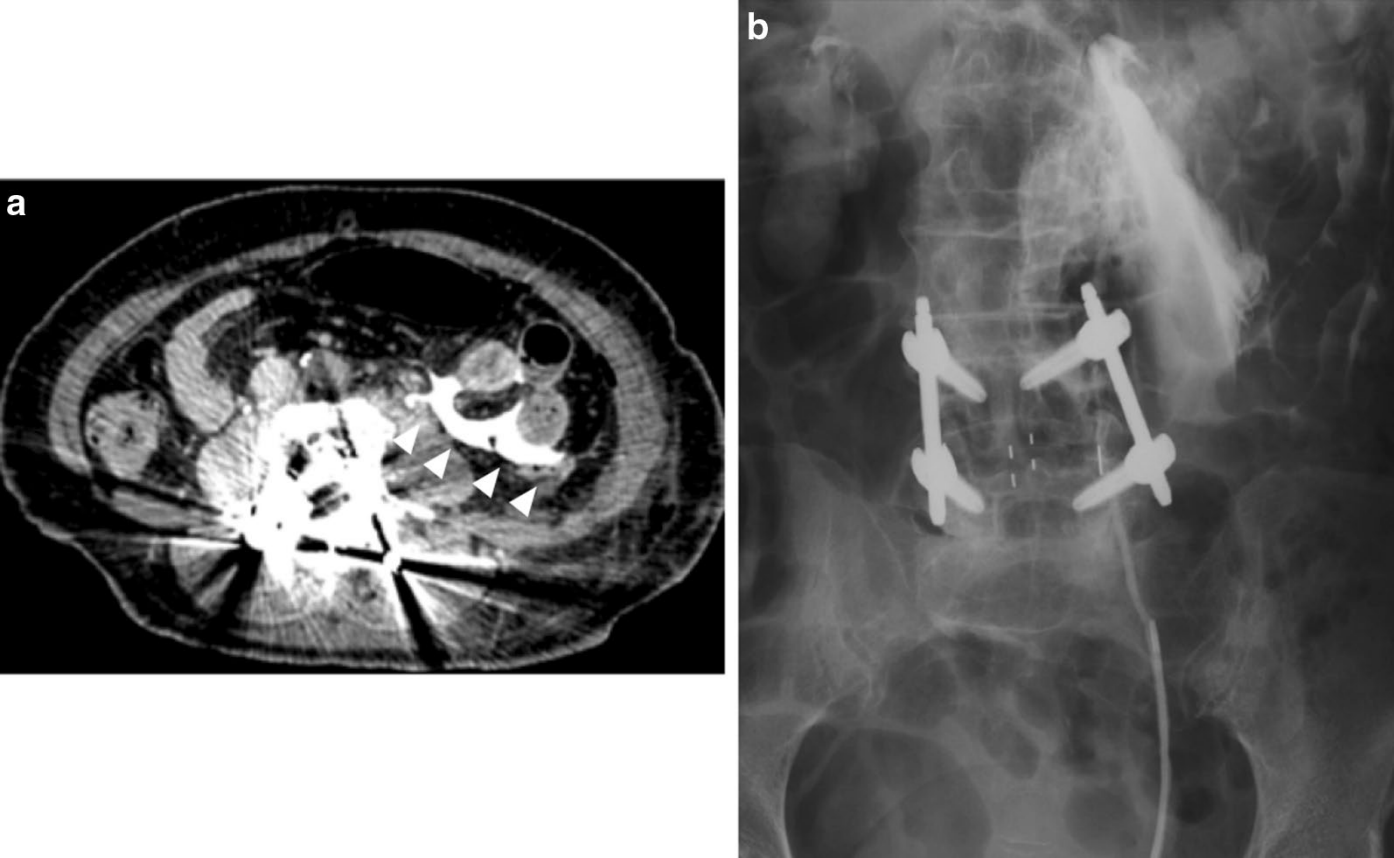
**a** Delayed-phase enhanced abdominal computed tomography scan at the L4/L5 level shows fluid collection in the left abdominal cavity extending from the left peritoneal space to the posterior iliopsoas region (*arrowheads*). **b** Left retrograde urography shows extravasation of contrast medium at the L4/5 level

Two days after the surgery, the patient complained of abdominal pain with apparent rebound tenderness. Closed physical examination and delayed contrast-enhanced computed tomography (CT) of the abdomen revealed fluid collection in the left abdominal cavity that extended from the peritoneal space to the posterior iliopsoas region (Fig. 3a). Furthermore, retrograde urography revealed a contrast leak at the level of the superior aspect of the L4 vertebra (Fig. 3b). These findings suggested ureteral injury. Continuous percutaneous nephrostomy (PNS) was performed after we unsuccessfully attempted retrograde ureteral stenting to attenuate symptoms. After the PNS was removed, double J-shaped ureteral stenting was successfully performed. Three months after stenting, the ureteral stent was removed with some ureteral stenosis, which required repeated ureteral dilatation and stenting.

One year after surgery, the patient is free from the preoperative symptoms with good postoperative radiological results and no urinary symptoms, however she still requires periodic changing of ureteral stents.

## Discussion

We encountered a case of ureteral injury following the OLIF procedure. It is a rare complication with a lower incidence than in other surgeries, including gynecologic surgery, pelvic surgeries for colon and rectal pathology, and vascular surgery [6–8]. Ureteral injury itself can occur following both traditional anterior lumbar surgeries and posterior lumbar surgeries [9–14], although there are few reports of its occurrence following minimally invasive OLIF surgery. We suspected that the patient had the abdominal pain with apparent rebound tenderness due to a ureteral injury. Thus, we diagnosed the injury using delayed contrast-enhanced CT and retrograde urography.

This complication can occur because of the limited exposure of the retroperitoneal space. Surgeons should be careful and sensitive to its incidence, as delaying the diagnosis of this injury could result in the need for nephrectomy [15, 16]. The injury is often difficult to detect because symptoms can be non-specific and occur immediately after surgery or later. However, symptoms including abdominal pain, fever, vomiting, and prolonged ileus, should be considered as signs of possible ureteral injury [17]. The current case may have led to a critical situation, including possible peritonitis by intra-abdominal urine leakage with possible future nephrectomy, but such a situation was avoided because we performed delayed contrast-enhanced CT [18].

Anatomically, ureters are located anterolaterally to the psoas muscle in the retroperitoneal space and next to the great vessels adjacent to the aspect of the disc space lying within a fat tissue envelope close to the posterior peritoneum. Ureters typically lie anterior to the spinal column with the swept peritoneum, and ureter injury rarely occurs. In the current case, the ureter may have been accidentally injured by the thread pin, which had slipped anteriorly, and not because of anatomical reasons. Another reason for the incident could have been the patient’s primary disease, RA, which can alter the properties of the soft tissue; such an alteration could have caused the ureter to remain in the trajectory of the fixation pin where it otherwise would not have been located [19].

## Conclusion

Ureteral injury can occur after OLIF surgery, although it has a low incidence and is sometimes iatrogenic. Surgeons should use caution when exposing the retroperitoneal space to avoid damage to minor organs, including the ureters, as well as to major organs. Delayed contrast-enhanced CT and retrograde urography are useful for diagnosing the injury.

## Abbreviations

OLIF: oblique lateral interbody fusion
RA: rheumatoid arthritis
CT: computed tomography
PNS: percutaneous nephrostomy
